# MiR-665 Regulates Vascular Smooth Muscle Cell Senescence by Interacting With LncRNA GAS5/SDC1

**DOI:** 10.3389/fcell.2021.700006

**Published:** 2021-07-27

**Authors:** Tianbin Chen, Qingyang Liang, Jialin Xu, Yanan Zhang, Yi Zhang, Liping Mo, Li Zhang

**Affiliations:** ^1^Functional Experiment Center, School of Basic Medical Sciences, Guangzhou Medical University, Guangzhou, China; ^2^Department of Cardiology, The First Affiliated Hospital of Guangdong Pharmaceutical University, Guangzhou, China; ^3^Key Laboratory of Animal Virology of Ministry of Agriculture, Center for Veterinary Sciences, Zhejiang University, Hangzhou, China

**Keywords:** vascular smooth muscle cells, LncRNA GAS5, miR-665, SDC1, senescence, atherosclerosis

## Abstract

**Background:** Vascular aging is considered a special risk factor for cardiovascular diseases, and vascular smooth muscle cells (VSMCs) play a major role in aging-related vascular remodeling and in the pathological process of atherosclerosis. Recent research has reported that long non-coding RNA/microRNA (lncRNA/miRNA) is a critical regulator of cellular senescence. However, the role and mechanism of lncRNA GAS5/miR-665 axis in VSMC senescence remain incompletely understood.

**Methods:** Cellular senescence was evaluated using senescence-associated β-gal activity, the NAD+/NADH ratio, and by immunofluorescence staining of γH2AX immunofluorescence. Differentially expressed miRNAs (DEMs) were identified by miRNA microarray assays and subsequently validated by quantitative real-time PCR (qRT-PCR). A dual luciferase reporter assay was conducted to confirm the binding of lncRNA GAS5 and miR-665 as well as miR-665 and syndecan 1 (SDC1). Serum levels of miR-665, lncRNA GAS5, and SDC1 in 93 subjects were detected by qRT-PCR. The participants were subdivided into control, aging, and early vascular aging (EVA) groups, and their brachial-ankle pulse wave velocity (baPWV) was measured.

**Results:** A total of 20 overlapping DEMs were identified in young and old VSMCs via microarray analysis. MiR-665 showed a significant alteration and, therefore, was selected for further analysis. Upregulation of miR-665 was found in aging VSMCs, and downregulation of miR-665 caused an inhibition of VSMCs senescence. Subsequently, the dual luciferase reporter assay determined the binding site of miR-665 with the 3′-UTR of lncRNA GAS5 and SDC1. Increased expression of lncRNA GAS5 expression inhibited the miR-665 level and VSMC senescence. However, as shown in rescue experiment results, either miR-665 overexpression or SDC1 knockdown significantly reversed the effects of lncRNA GAS5 on VSMC senescence. Finally, compared with that of the control group, miR-665 was highly expressed in serum samples in the aging and EVA groups, especially in the EVA groups. On the contrary, serum levels of lncRNA GAS5 and SDC1 were lower in these two groups. Collectively, in the aging and EVA groups, miR-665 expression was negatively correlated with lncRNA GAS5 and SDC1 expression.

**Conclusion:** miR-665 inhibition functions as a vital modulator of VSMC senescence by negatively regulating SDC1, which is achieved by lncRNA GAS5 that sponges miR-665. Our findings may provide a new treatment strategy for aging-related cardiovascular diseases.

## Introduction

Cardiovascular disease (CVD) is the main factor contributing to a burden for the elderly, accounting for 30.3% of the total burden of disease in people aged 60 and above (Prince et al., 2015). Aging, particularly vascular aging, is considered the major non-modifiable risk factor in the development of CVD. The morphological and functional features of aging changes in blood vessels are vascular remodeling and stiffness (Zhang and Tao, 2018), and pulse wave velocity (PWV) is the gold standard method for evaluating central vascular stiffness (Raij and Gonzalez-Ochoa, 2011; Zhang and Tao, 2018). Vascular smooth muscle cells (VSMCs), the most abundant intrinsic cells in the vessel wall, play a key role in vascular aging. Aging-related vascular remodeling is initially caused by the transition of VSMC from a contractile to a secretory phenotype (Uryga and Bennett, 2016; Stojanovic et al., 2020). VSMC senescence leads to cell dysfunction and promotes the occurrence of aging-related CVD, such as atherosclerosis and hypertension, whereupon inhibition of VSMC senescence may be one of the treatments for aging-related CVD.

Lately, the field of non-coding RNA has received increasing attention, especially long non-coding RNAs (lncRNAs) and microRNAs (miRNAs). In recent years, these non-coding RNAs have been reported to play a crucial role in the regulation of CVD, such as atherosclerosis and hypertension (Huang, 2018; Prestes et al., 2020), and are closely related to aging (Li et al., 2018; Cao et al., 2020; Zhao et al., 2020). In our previous study, miRNA microarray assays identified that miR-665 was the most significantly differentially expressed miRNA (DEM) in aging VSMCs, and it is a vital modulator of VSMC senescence (Zhang et al., 2020). LncRNA, as a miRNA sponge, negatively regulates the regulation of miRNA on downstream target genes through the mechanism of competitive endogenous RNA (ceRNA) (Greco et al., 2019; Zhao et al., 2019). LncRNAs/miRNAs are novel cellular senescence regulators. For example, Tan P et al. find that lncRNA-ANRIL inhibits VSMC senescence by regulating miR-181a (Tan et al., 2019). Consequently, we were looking for a lncRNA that may target miR-665 and may be related to VSMC senescence. LncRNA growth-specific inhibitor 5 (lncRNA GAS5) is related to cell proliferation, which contributes to the regulation of mammalian cell cycle and apoptosis (Tang et al., 2019). However, whether lncRNA GAS5 directly targets miR-665 and whether lncRNA GAS5/miR-665 axis functions as an important modulator in VSMC senescence are still not fully understood.

In the present study, we used replicative senescent VSMCs to investigate a novel miR-665 signaling pathway that interacts with lncRNA GAS5 and syndecan 1 (SDC1), contributing to an improvement in cell senescence. Our study provides an experimental basis for investigating the mechanism by which lncRNA GAS5/miR-665 axis regulates VSMC senescence.

## Methods and Materials

### Cell Culture and Cell Transfection

Human aorta VMSCs (HA-VSMCs, No: CRL-1999) were purchased from the American Type Culture Collection (ATCC; Manassas, VA, USA) and cultured in Dulbecco's modified Eagle medium (DMEM; Sigma-Aldrich, St. Louis, MO, USA) containing 10% FBS (Gibco; Thermo Fisher Scientific, Inc., Waltham, MA, USA) at 37°C with 5% CO_2_. The medium was renewed every 2 to 3 days. HA-VSMCs passage 5 (young) and passage 15 (aging) with 70–90% confluence were used.

MiR-665 inhibitor/mimics, small interfering RNA targeting SDC1 (siSDC1), and the negative control oligonucleotides (miR-NC and siNC) were synthesized by RiboBio Co., Ltd. (Guangzhou, China). The pcDNA3.1/lncRNA GAS5 vector was constructed by OriGene Technologies, Inc. (Beijing, China). In six-well plates, each well was plated with 2 × 10^5^ VMSCs in triplicate and then incubated overnight prior to transfection. All plasmid transfections were performed using Lipofectamine™ 2000 (Invitrogen, USA) according to the provided instructions.

### RNA Extraction

Total RNA, including miRNA from cells and serum, was extracted by using TRIzol reagent (Invitrogen, Carlsbad, CA, USA) and miRNeasy Serum/Plasma kit (QIAGEN, #217184), according to the manufacturer's protocol. The quantity of RNA in all samples was measured with a NanoDrop 2000c (Thermo Fisher Scientific).

### MiRNA Microarray

The total RNA extracted from cells was further purified using an RNeasy Mini Spin Column Kit (Qiagen, Inc., Valencia, CA, USA). After being checked for quality, the RNA samples were labeled using reagents in a miRCURY™ Power Labeling Kit (Exiqon, Denmark) according to the manufacturer's instructions. The labeled RNA samples were hybridized onto a miRCURYTM LNA Array (Exiqon, Denmark), washed with a Wash Buffer Kit (Exiqon, Denmark) and then scanned with a GenePix 4000B microarray scanner (Axon Molecular Devices, San Jose, CA, USA).

### Microarray Data Analysis

Raw data extraction was performed with GeneChip Command Console software (version 4.0; Affymetrix, Inc., Santa Clara, CA, USA). After normalization, DEMs in the two groups (young and aging cells) were identified by using specific cutoff criteria (*P* < 0.05 and fold-change >2.0). Hierarchical clustering was performed to display the DME, and then a VENN analysis for screening the overlapping DME. Subsequently, the target genes of the overlapping DEM were predicted by the TargetScan database (http://targetscan.org/). These putative target genes were then subjected to Gene Ontology (GO) and Kyoto Encyclopedia of Genes and Genomes (KEGG) pathway analysis. The significant GO and KEGG pathway terms were identified by using a cutoff *P* < 0.05 and a count >2. Data was stored in the GEO database (https://www.ncbi.nlm.nih.gov/geo/query/acc.cgi?acc=GSE174451).

### Luciferase Reporter Assay

The lncRNA GAS5 and SDC1 3′UTR segments containing the putative binding site for miR-665 were amplified and inserted into a pmirGLO vector (Promega), resulting in the lncRNA GAS5 WT and SDC1 WT. Meanwhile, a mutation was introduced into the potential miR-665 binding sites (designated as lncRNA GAS5 MUT and SDC1 MUT) by using the Quick Change Stratagene method. VSMCs (2 × 10^4^ per well) were cultured in 24-well plates and then transfected with 50 nM miR-665 mimic/inhibitor together with 0.5 μg of luciferase reporter vector containing the GAS5 WT/MUT or SDC1 WT/MUT, by using Lipofectamine 2000. Forty-Eight hours later, luciferase activity was assessed with a Dual-Luciferase Reporter Assay System (Promega).

### Senescence-Associated β-Galactosidase Activity Assay

Cellular senescence was analyzed using a senescence-associated β-galactosidase (SA-β-gal) staining kit (Beyotime Biotechnology) according to the manufacturer's protocol. Briefly, HA-VSMCs in six-well plates were grown to 50% confluence and fixed with β-gal fixation solution (2% formaldehyde/0.2% glutaraldehyde in PBS) for 5 min, washed with PBS, and then incubated overnight with SA-β-gal staining solution at 37°C. The percentage of SA-β-gal-positive cells was examined in four randomly selected fields under a microscope (Leica Microsystems GmbH, Weltzar, Germany) and calculated as previously described (Tan et al., 2016).

### NAD+/NADH Assay

The cellular content of NAD^+^ and NADH was determined using a NAD+/NADH Assay Kit with WST-8 (S0175, Beyotime Biotechnology, Shanghai, China) according to the manufacturer's instructions. In brief, approximately 1 × 10^6^ VMSCs were plated into each well of six-well plates and incubated with 200 μLof NAD+/NADH extraction solution. The NAD+/NADH ratio was calculated based on the NAD+ and NADH standard curves.

### γ-H2AX and GAS5 Immunofluorescence Assay

VSMCs were plated in six-well plates. After interventions and cell transfection and being cultured overnight, VSMCs were fixed with 4% paraformaldehyde, and after 0.5% tritonX-100 infiltration, 5%BSA working solution was utilized for the blocking step at ambient temperature for 2 h. After that, the cells were incubated with γ-H2AX antibody and GAS5 antibody at 4°C overnight, followed by an incubation with Alexa Fluor 594 dye-conjugated secondary antibodies at ambient temperature in the dark for 2 h. Next, the samples were stained with DAPI at ambient temperature for 10 min. Finally, the slides were photographed under an Olympus confocal microscope (FV1000MPE, Olympus, Tokyo, Japan).

### Quantitative Real-Time PCR Assay

QRT-PCR was performed using a PrimeScript^TM^ RT reagent kit with gDNA Eraser (TaKaRa, Dalian, China) following the manufacturer's protocol. The levels of mRNA expression were quantified by standard real-time PCR protocol with SYBR Premix Ex Taq (TaKaRa, Dalian, China). All reactions were carried out in triplicate. GAPDH was used as a reference gene. The primers used were as follows: lncRNA GAS5, F: GTTGTGTCCCCAAGGAAGGATG AG, R: TGTCTAATGCCTGTGTGCCAATGG; miR-665, F: ACACTCCAGCTGGGACCAGGAGGCTGAG, R: CTCAACTGGTGTCGTGGA; SDC1, F: AAGATATCACCTTGTCACAGCA, R: GTTCTGGAGACGTGGGAATAG; U6, F: CTCGCTTCGGCAGCACA, R: AACGCTTCACGAATTTGCGT; GAPDH, F: CAGGAGGCATTGCTGATGAT, R: GAAGGCTGGGGCTCATTT.

### Western Blot Analysis

Briefly, total protein was extracted using a RIPA buffer (Beyotime Institute of Biotechnology, Shanghai, China), and the protein concentration in each sample was determined with a BCA assay kit (Beyotime Institute of Biotechnology). Next, 30 μg protein from each sample was separated by 10% SDS-PAGE and transferred onto PVDF membranes. The membranes were subsequently blocked with 5% non-fat milk at ambient temperature for 1 h. After that, co-incubation of the membranes and primary antibodies anti-γ-H2AX, anti-SDC1, and anti-GAPDH (Abcam, Cambridge, UK) was carried out at 4°C overnight, followed by another 2 h incubation with the corresponding horseradish peroxidase-conjugated secondary antibodies at ambient temperature. The protein bands were visualized by using an Enhanced Chemiluminescent Substrate kit (Pierce; Thermo Fisher Scientific, Inc.) and analyzed by Image-Pro Plus 6.0.

### Clinical Serum Samples

Study participants were recruited from 93 individuals aged 24–79 years, including males and females, who underwent health examinations at the First Affiliated Hospital of Guangdong Pharmaceutical University (Guangzhou, China) from July 2019 to September 2020. The experiment was carried out with the informed content from each participant and an approval by the Clinical Research Ethics Committee of the First Affiliated Hospital of Guangdong Pharmaceutical University.

The exclusion criteria were as follows: definite CVD, such as coronary heart disease, heart failure, rheumatic heart disease; diabetes mellitus; being treated for hypertension; being treated for lipid regulation; acute or chronic inflammatory diseases; tumors or diseases of the hematopoietic system; and drug or alcohol abuse. Smoking and untreated dyslipidemia had no significant effect on PWV, and therefore, the subjects with these two cardiovascular risk factors could be included.

Serum samples were isolated by centrifugation (1,200 × g, 10 min) followed by another centrifugation (10,000 × g, 10 min) to remove residual debris. All centrifugations were performed at 4°C, and serum samples were stored at −80°C for RNA extraction.

### PWV Measurement

Brachial-ankle pulse wave velocity (baPWV), a marker of arterial stiffness, was measured using an automatic waveform analyzer (modelBP-203RPEIII, OMRON, Japan). The included subjects were subdivided into three groups according to baPWV and age: control, early vascular aging (EVA), and aging groups. Two standard deviations of age-corrected PWV values greater than the normal reference values of healthy people are usually used as EVA criteria.

### Animals

Male Sprague–Dawley rats, aged 6 (young group) and 24 months (old group), were purchased from Guangdong Medical Laboratory Animal Center (Guangzhou, China). All rats were caged under temperature controlled conditions with water and food *ad libitum*. The experimental procedures and protocols were approved by the Institutional Animal Care and Use Committee of Guangdong Pharmaceutical University. The media of aortic tissue isolated from young and old rats were collected as previously described (Wang and Lakatta, 2002). Then, the content of miR-665 was detected by qRT-PCR.

### Statistical analysis

SPSS version 13.0 for Windows (SPSS Inc., IL, USA) and GraphPad Prism 4.0 (GraphPad Software, CA, USA) were utilized for data statistics. The difference between two groups was analyzed by Student's *t*-test, and one-way analysis of variance (ANOVA) for analyzing among more than two groups, followed by a Bonferroni test. The correlation analysis between miR-665 and lncRNA GAS5 as well as SDC1 in the serum was conducted using Pearson correlation test. A *P* < 0.05 was considered to be statistically significant.

## Results

### MiR-665 Is Involved in the Regulation of VSMC Senescence

**Figure 1:**
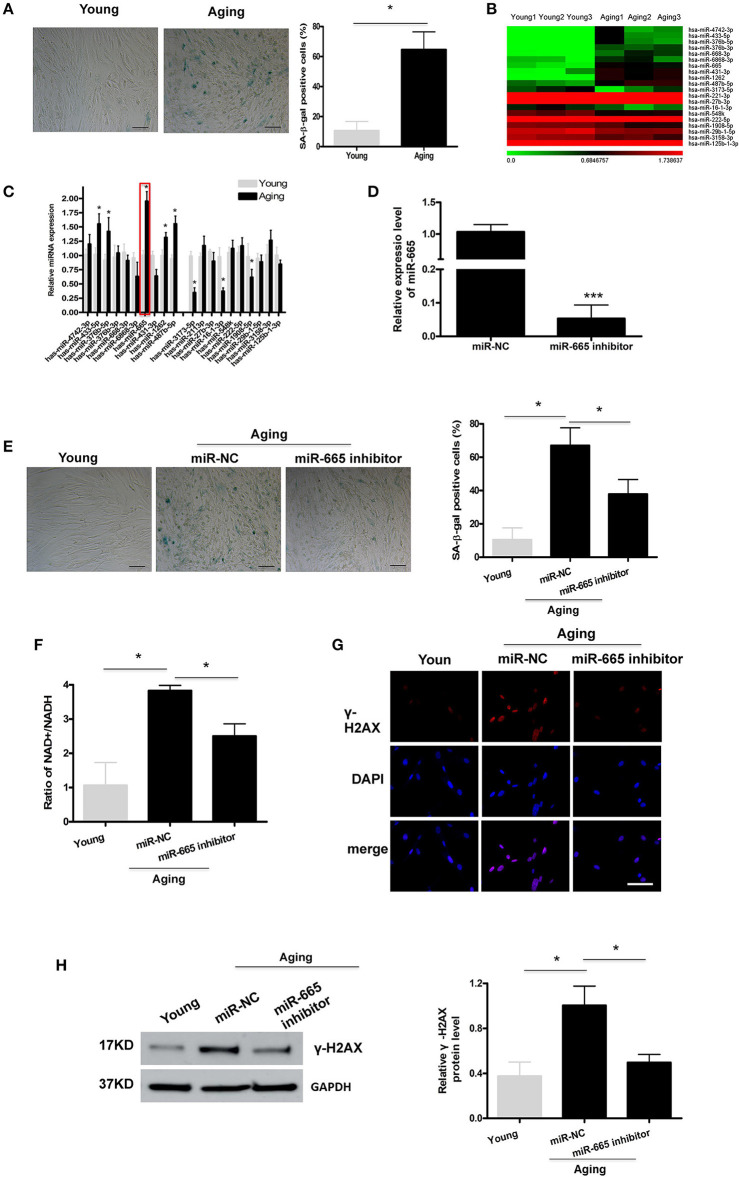
MiR-665 is involved in the regulation of VSMC senescence. **(A)** Representative photomicrographs and average data of SA β-gal staining (blue) in young and aging VSMCs. Scale bar indicates 50 μm. *P < 0.05; Original magnification × 100. **(B,C)** Top 20 DEMs in young and aging VSMCs as determined via microarray analysis and qRT-PCR assays. *P < 0.05 compared with young groups. **(D)** qRT-PCR-based determination of miR-665 inhibition efficiency in aging VSMCs. ***P < 0.001. **(E)** Representative photomicrographs and average data of SA β-gal staining in young VSMCs and aging VSMCs transfected with miR-665 inhibitor or miR-NC. Scale bar indicates 50 μm. *P < 0.05; Original magnification × 100. **(F)** Representative average ratio of NAD+/NADH in young VSMCs and aging VSMCs transfected with miR-665 inhibitor or miR-NC. *P < 0.05. **(G)** Representative immunofluorescence assay of γ-H2AX in young and aging VSMCs transfected with miR-665 inhibitor or miR-NC. *P < 0.05; Scale bar indicates 20 μm. **(H)** Representative Western blot assays of γ-H2AX in young and aging VSMCs transfected with miR-665 inhibitor or miR-NC. *P < 0.05.

To explore the molecular mechanism by which miRNAs involve in cellular senescence, a cellular senescence model was established by passaging HA-VSMCs. As shown in Figure 1A, SA-β-gal staining analysis showed that the positive rate of SA-β-gal was increased significantly in aging cells. Then miRNA microarray was used to analyze the DEMs in young and aging VSMCs, and we identified the top 20 overlapping DEMs, followed by the validation by qRT-PCR (Figures 1B,C; Supplementary Table 1). The qRT-PCR results revealed that, in aging VSMCs, the levels of miR-433-5p, miR-376b-5p, miR-665, miR-1262, and miR-487b-5p expression were significantly upregulated, and the levels of miR-3173-5p, miR-16-1-3p, and miR-1908-5p expression were markedly downregulated. Our previous study pointed out miR-665 as a novel regulator of VSMC senescence. Most importantly, miR-665 showed a more obvious alteration than other miRNAs in miRNA microarray and qRT-PCR assays; miR-665 was, therefore, selected for further analysis.

Next, we investigated whether miR-665 was involved in VSMCs senescence. Aging VSMCs were transfected with miR-665 inhibitor, and the transfection efficiency was determined using qRT-PCR (Figure 1D). γ-H2AX could be utilized to explore DNA damage and repair (Sharma et al., 2012). Cells would have a lower NAD+/NADH ratio after mitochondrial dysfunction-aging (Wiley et al., 2016). As shown in Figures 1E–H, the increases in numbers of SA-β-gal-positive cells, NAD+/NADH ratio, γ-H2AX immunostaining, and protein in the aging group were significantly reversed by miR-665 inhibitor.

### lncRNA GAS5 Acts as a Molecular Sponge to Negatively Regulate miR-665

**Figure 2:**
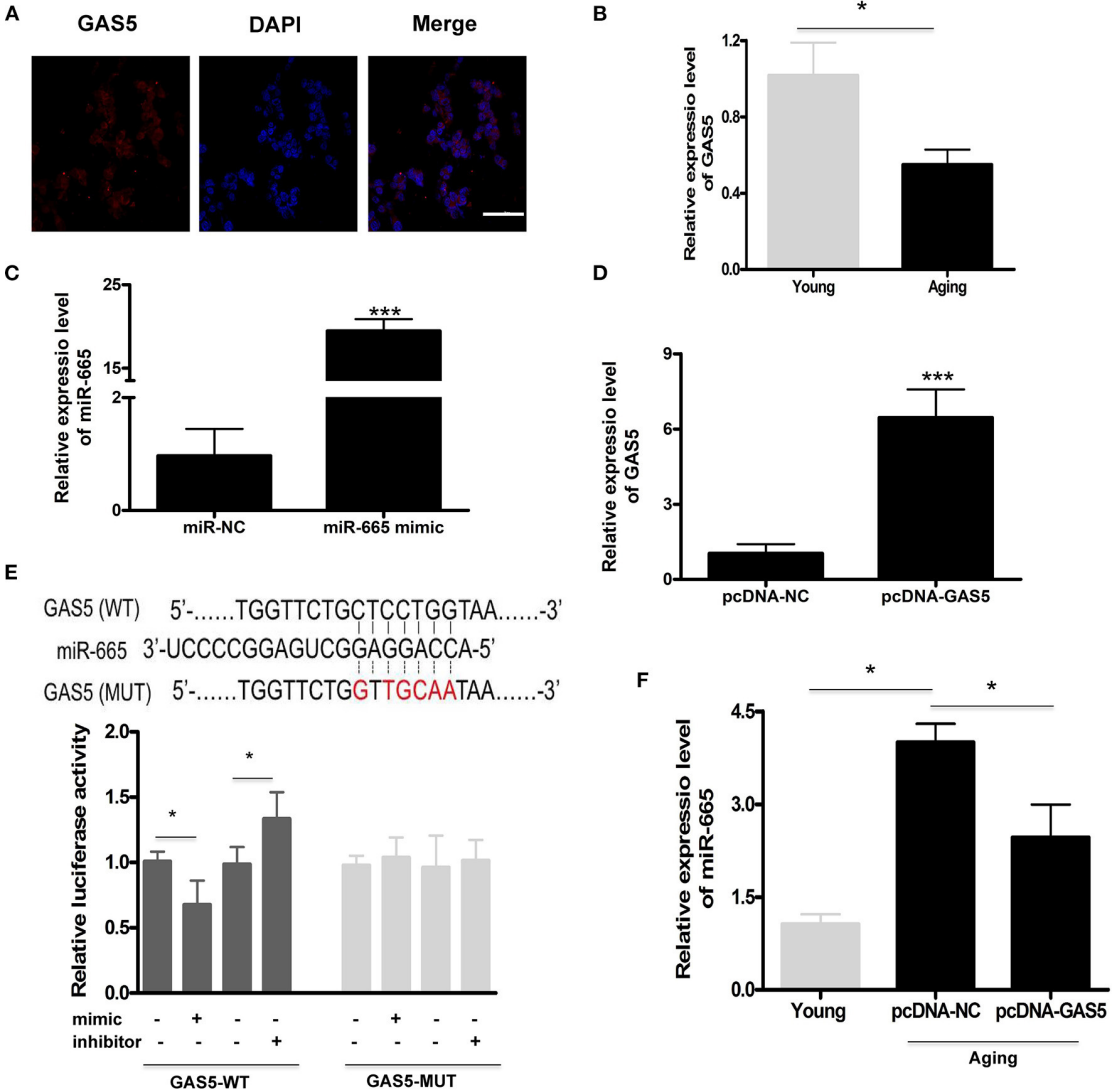
LncRNA GAS5 acts as a molecular sponge to negatively regulate miR-665. **(A)** Representative subcellular fractionation of lncRNA GAS5. Scale bar indicates 20 μm. **(B)** Representative average data of lncRNA GAS5 determined by qRT-PCR assays in young and aging VSMCs. *P < 0.05. **(C)** qRT-PCR-based determination of transfection efficiency of miR-665 in aging VSMCs. ***P < 0.001. **(D)** qRT-PCR-based measurement of transfection efficiency of lncRNA GAS5 in aging VSMCs. ***P < 0.001. **(E)** Representative predicted binding region of miR-665 and lncRNA GAS5, and relative luciferase activity determined by dual luciferase reporter assay. *P < 0.05. **(F)** Representative average data of miR-665 determined by qRT-PCR in young and aging VSMCs transfected with pcDNA-GAS5 and pcDNA-NC. *P < 0.05.

We found that miR-665 could regulate VSMC senescence, but the molecular mechanism remained unclear. Previous studies show that lncRNAs can act as miRNA sponges, thus reducing regulatory effects of miRNAs on mRNAs (Paraskevopoulou and Hatzigeorgiou, 2016). Here, we explored the potential relationship between lncRNA GAS5 and miR-665. First, we determined the subcellular fractionation of lncRNA GAS5 and found that lncRNA GAS5 was mainly located in the cytoplasm, suggesting its role in post-transcriptional control (Figure 2A). Next, we examined the expression of lncRNA GAS5 in young and aging VSMCs and found the significant downregulation of its expression in aging VSMCs (Figure 2B).

Subsequently, aging VSMCs were transfected with miR-665 and lncRNA GAS5, and the transfection efficiency was determined using qRT-PCR (Figures 2C,D). As shown in Figure 2E, online bioinformatics tools (TargetScan software) showed that a potential miR-665 binding site was identified on the lncRNA GAS5 3′-UTR, which was further validated by luciferase gene reporter analysis. With transfection of miR-665 mimics/inhibitor, the relative luciferase activity significantly decreased/increased in wild type but did not change in the MUT type (Figure 2E).

Finally, we used pcDNA-GAS5 to overexpress lncRNA GAS5 in aging VSMCs, leading to a significant decrease of miR-665 expression. Results showed that the expression of miR-665 was significantly decreased when transfected with pcDNA-GAS5 (Figure 2F).

### SDC1 Functions as a Target of miR-665 in VSMCs

**Figure 3:**
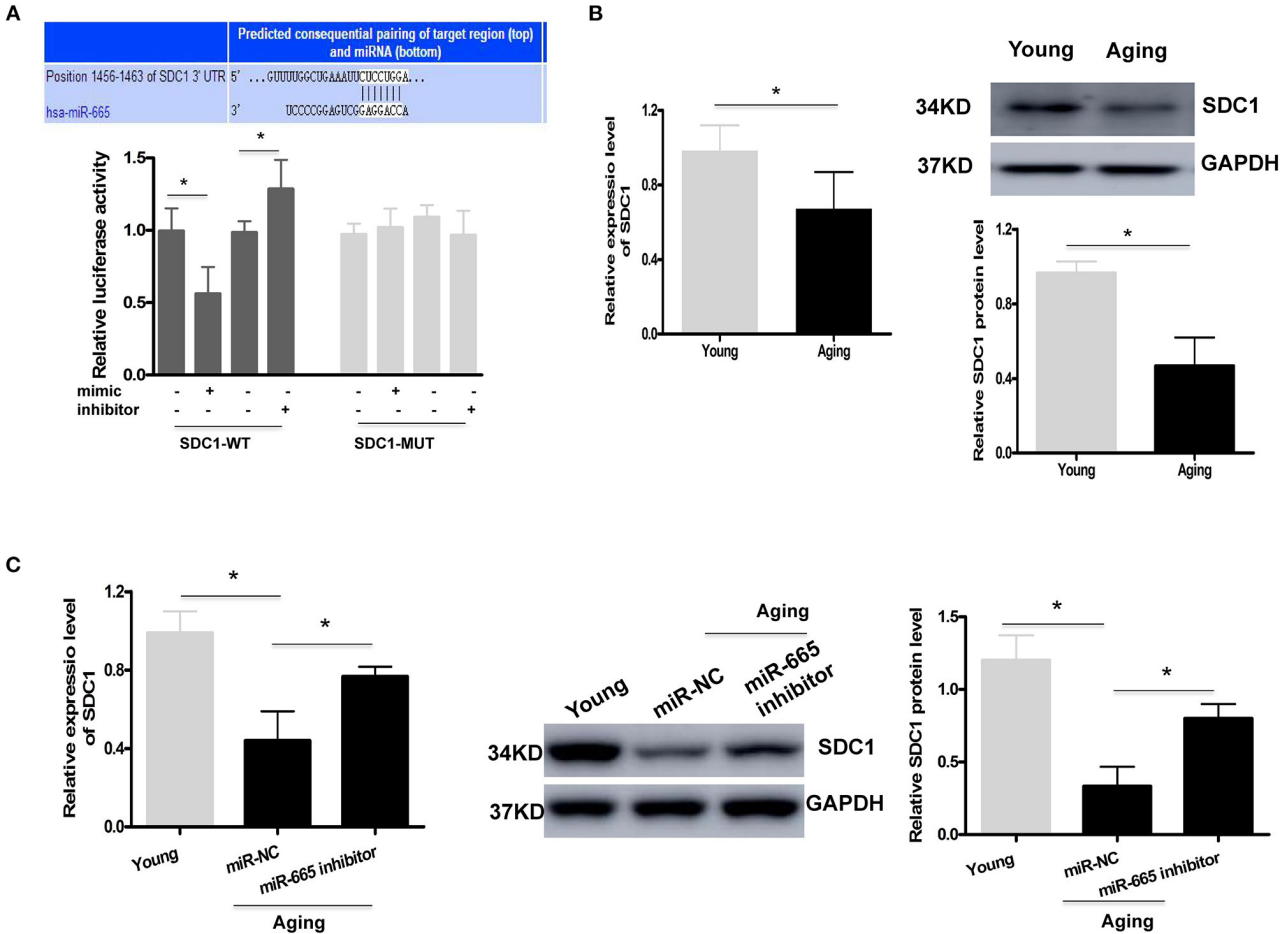
SDC1 functions as a target of miR-665 in VSMCs. **(A)** Representative predicted binding region of miR-665 and SDC1 and relative luciferase activity determined by dual luciferase reporter assay. *P < 0.05. **(B)** Representative qRT-PCR and Western blot assays of SDC1 in young and aging VSMCs. *P < 0.05. **(C)** Representative qRT-PCR and Western blot assays of SDC1 in young and aging VSMCs transfected with miR-665 inhibitor or miR-NC. *P < 0.05.

As with other miRNAs, miR-665 can negatively regulate gene expression by binding to the 3′UTR of downstream target genes. A bioinformatics analysis was performed to identify the potential target genes of miR-665. As shown in Figure 3A, a potential binding site to miR-665 was identified on the 3′UTR of SDC1 mRNA. Therefore, a luciferase reporter assay was conducted to further examine whether miR-665 might regulate SDC1 expression. Our results show that the relative luciferase activity in the SDC1 WT was significantly downregulated/upregulated by transfection with the miR-665 mimic/inhibitor although there was no obvious change in the SDC1 MUT. We then analyzed the expression of SDC1 mRNA and protein in young and aging VSMCs. As expected, SDC1 mRNA and protein levels in aging VSMCs were all significantly lower than those in young VSMCs (Figure 3B). We also detected the expression of SDC1 in the transcription and translation levels transfecting with miR-665 inhibitor. Consequently, inhibition of miR-665 markedly increased SDC1 protein and mRNA levels in aging VSMCs (Figure 3C).

### MiR-665 Interacts With lncRNA GAS5 and SDC1 to Regulate VSMC Senescence

**Figure 4:**
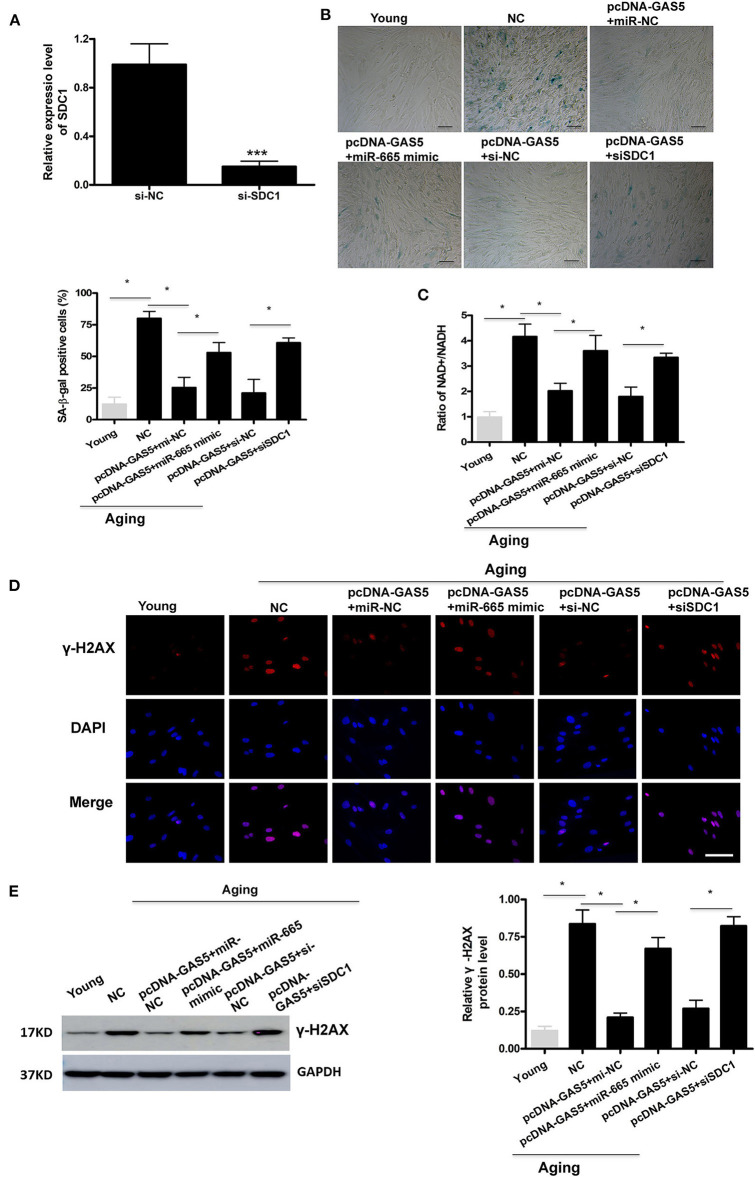
MiR-665 interacts with lncRNA GAS5 and SDC1 to regulate VSMC senescence. **(A)** qRT-PCR-based evaluation of transfection efficiency of SDC1 in aging VSMCs. ***P < 0.001. **(B)** Representative photomicrographs and average data of SA β-gal staining in young and aging VSMCs cotransfected with pcDNA-GAS5 and miR-665 mimic/miR-NC or si-SDC1/si-NC. *P < 0.05. **(C)** Representative average ratio of NAD+/NADH in young and aging VSMCs cotransfected with pcDNA-GAS5 and miR-665 mimic/miR-NC or si-SDC1/si-NC. *P < 0.05. **(D)** Representative immunofluorescence assay of γ-H2AX in young and aging VSMCs cotransfected with pcDNA-GAS5 and miR-665 mimic/miR-NC or si-SDC1/si-NC. *P < 0.05. **(E)** Representative Western blot assays of γ-H2AX in young and aging VSMCs cotransfected with pcDNA-GAS5 and miR-665 mimic/miR-NC or si-SDC1/si-NC. *P < 0.05.

First, we successfully transfected si-SDC1 into aging VSMCs (Figure 4A). Then, rescue experiments were performed to further confirm the regulation of lncRNA GAS5/miR-665/SDC1 to VSMC senescence. As shown in Figure 4B, the rate of SA-β-gal positive cells was greatly decreased by overexpression of lncRNA GAS5 in aging VSMCs, which was restored by miR-665 overexpression. SA-β-gal results also demonstrate that the rate of SA-β-gal positive cells was significantly increased by co-transfection with pcDNA-GAS5 and SDC1 siRNA, compared with corresponding control siRNA (Figure 4B). As expected, miR-665 overexpression or SDC1 silencing remarkably reversed the effects of lncRNA GAS5 on the NAD+/NADH ratio, γ-H2AX immunostaining, and protein (Figures 4C–E).

### Serum Level of miR-665 Is Higher and Negatively Correlated to lncRNA GAS5 and SDC1 in the Aging and EVA Groups

**Figure 5:**
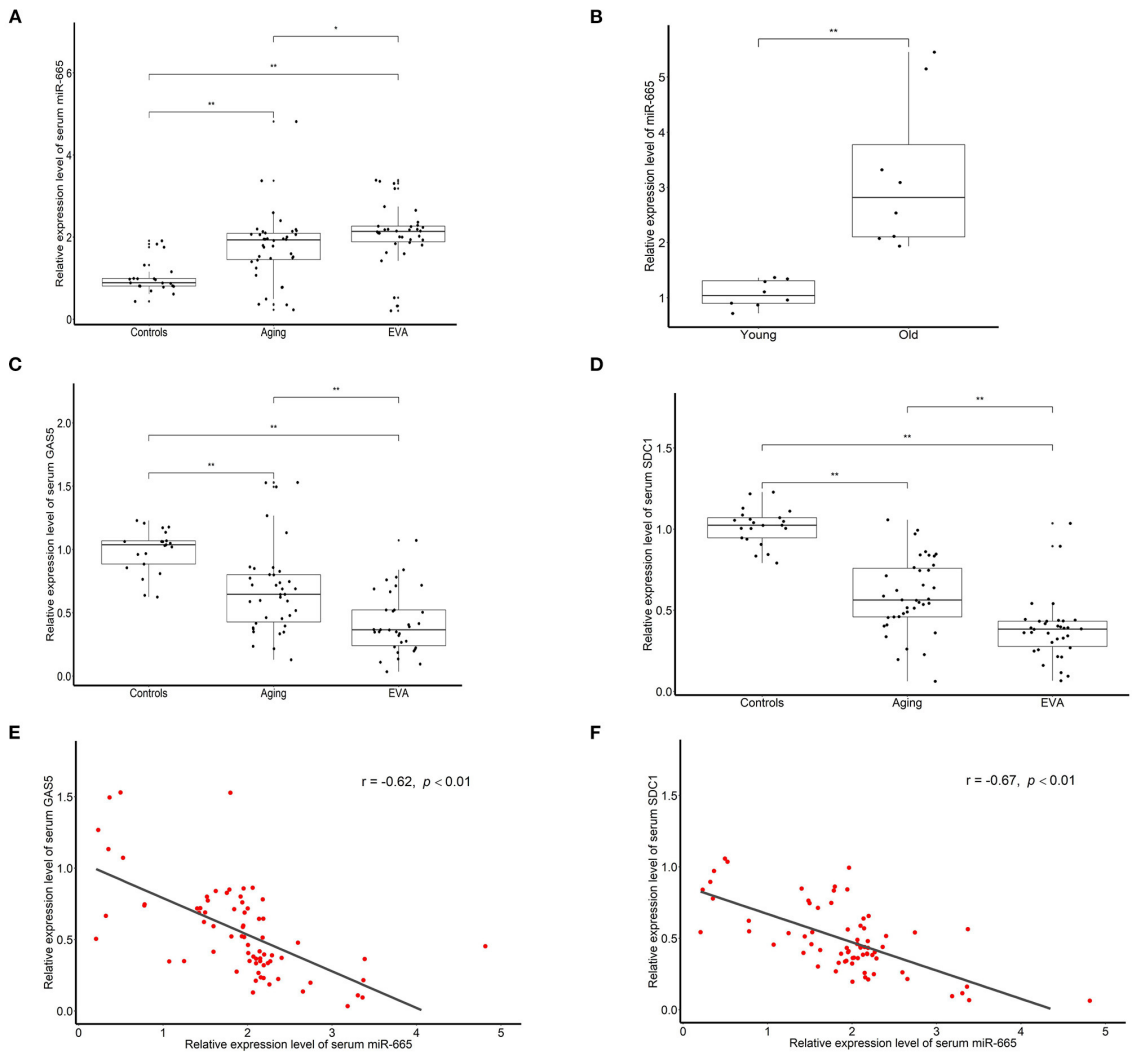
Serum level of miR-665 is higher and is negatively correlated to lncRNA GAS5 and SDC1 in the aging and EVA groups. **(A)** qRT-PCR assays of miR-665 for serum samples of aging, EVA, and control individuals. *P < 0.05, **P < 0.01. **(B)** qRT-PCR assays of miR-665 in the media of central arteries of young and old rats. **P < 0.01. **(C)** qRT-PCR assays of lncRNA GAS5 for serum samples of aging, EVA, and control individuals. **P < 0.01. **(D)** qRT-PCR assays of SDC1 for serum samples of aging, EVA, and control individuals. **P < 0.01. **(E)** Pearson correlation analysis of serum miR-665 and serum lncRNA GAS5 in individuals with aging and EVA. *r* = −0.62, *P* < 0.01. **(F)** Pearson correlation analysis of serum miR-665 and serum SDC1 in individuals with aging and EVA. *r* = −0.67, *P* < 0.01.

To explore the effect of miR-665 in aging, we used qRT-PCR to detect the expression of miR-665 in serum samples from 38 aging and 34 EVA individuals and compared the results with those from 21 controls. We found that miR-665 were highly expressed in the aging and EVA group, especially in EVA individuals (Figure 5A). Indeed, in animal experiments, we found that miR-665 expression was markedly higher in the media of central arteries of old rats when compared with that of young rats (Figure 5B). At the same time, the expression of lncRNA GAS5 and SDC1 in the aging and EVA groups was lower than that of control group (Figures 5C,D). Similarly, their expression was lowest in the EVA group. The correlation analysis showed that the expression of miR-665 mRNA was negatively correlated with the expression of lncRNA GAS5 in 72 individuals with EVA and aging (*r* = −0.62, *P* < 0.01; Figure 5E). We also revealed a negative correlation between miR-665 mRNA and SDC1 mRNA in 72 individuals (*r* = −0.67, *P* < 0.01; Figure 5F).

## Discussion

The results of the present study demonstrate that miR-665 level was increased in aging VSMCs. In addition, VSMC senescence was attenuated by miR-665 inhibition. Moreover, miR-665 regulated VSMC senescence via interacting with lncRNA GAS5 and SDC1. Finally, in clinical serum samples, miR-665 was higher in individuals with aging and EVA, especially in EVA individuals. Collectively, lncRNA GAS5/miR-665/SDC1 may provide new insights into the pathogenesis of vascular aging.

Several studies demonstrate that the functional changes of miRNAs affect the expression of aging-related genes through regulating key molecular pathways (Kim et al., 2017; Majidinia et al., 2020). The aberrant expressions of miR-21, miR-155, miR-126, miR-146a/b, miR-143/145, miR-223, and miR-221 were found in aging-associated CVD (Gangwar et al., 2018). Although miRNAs have become the target of research on aging and aging-related interventions, the molecular mechanism by which miR-665 regulates VSMC senescence is not fully understood. In this study, a microarray analysis found a significant differential expression of miR-665 in young and aging VSMCs, and miR-665 could function as a vital modulator of VSMC senescence. Through experiments on animals and patients, we found that the serum level of miR-665 was higher in aging groups including EVA individuals compared with that of young controls. No related survey has been carried out on the direct relationship between miR-665 and vascular senescence. However, the abnormal expression of miR-665 in cardiomyocyte, cardiac and vascular endothelial cells, VSMCs, and its regulation on diseases indicate that miR-665 may play an important role in regulating cardiovascular function (Li et al., 2017; Fan et al., 2018; Lin et al., 2019; Yu et al., 2019; Liu et al., 2020). In addition, Fan et al. (2018) and Li et al. (2017) report that increased miR-665 resulted in decreased proliferation of vascular endothelial cells and VSMCs, supporting the possibility that miR-665 is involved in the process of vascular senescence.

LncRNA GAS5 is a kind of lncRNA related to cell proliferation, and in recent years, its regulatory roles in CVD has been paid more and more attention (Jiang and Ning, 2020). However, the expression of lncRNA GAS5 in aging-related diseases and its influence on aging are reported with different results. lncRNA GAS5 expression was increased in peripheral blood mononuclear cells of patients with type 2 diabetes mellitus, and it was positively correlated with aging markers (Sathishkumar et al., 2018). However, in an animal experiment, compared with young mice, the expression level of lncRNA GAS5 in the ovary of middle-aged mice was downregulated (Cuomo et al., 2018). Yao et al. (2019) demonstrate that knockdown of lncRNA GAS5 in vascular endothelial progenitor cells can significantly inhibit cell proliferation and stimulate cell senescence. In addition, lncRNA GAS5 knockdown can aggravate the microvascular dysfunction induced by hypertension, affecting VSMC phenotypic transformation (Wang et al., 2016). The above differences may be due to the fact that lncRNA GAS5 plays different roles in different cell types and diseases.

LncRNAs act as endogenous miRNA sponges to regulate miRNAs and, thus, regulates gene expression. The mechanism of ceRNA, namely, the RNA-miRNA regulatory pathway, has important biological significance. In the present study, double luciferase experiments confirmed that miR-665 could directly target lncRNA GAS5 3'-UTR. Furthermore, lncRNA GAS5 was downregulated and negatively regulated the expression of miR-665 in senescent VSMCs. In clinical experiments, we also found that the serum level of miR-665 was negatively correlated with lncRNA GAS5 in individuals with aging and EVA. In addition, increased miR-665 reversed the effects of lncRNA GAS5 on VSMC senescence, determining by SA-β-Gal staining, NAD+/NADH ratio, and immunostaining of γ-H2AX. The confirmed results are consistent with our previous prediction. Until now, no related literature has been found on the negative regulation of miR-665 by lncRNA GAS5 on cell senescence and aging. There is evidence that lncRNA GAS5 is associated with cell and tissue senescence through sponging miRNA (Yao et al., 2019; Cabiati et al., 2020).

Among the several potential target genes of miR-665 predicted by the TargetScan software, SDC1 was reported to be associated with senescence. Similarly, double luciferase experiments confirmed that only the relative luciferase activity in the SDC1 WT was significantly downregulated/upregulated by transfection with miR-665 mimic/inhibitor.

SDC1 is one of the crucial members of the heparin sulfate proteoglycan family on the cell surface. Oh et al. (2011) prove that the expression of SDC1 was decreased in aged skin in both males and females. Cells depleted of SDC1 exhibited typical senescence phenotypes (Kang et al., 2020). Chaterji et al. (2014) find that SDC1 knockout induced a contractile-to-secretory phenotype shift in VSMCs with a corresponding increase to inflammation. In the present study, the expression of SDC1 was decreased in senescent VSMCs at the transcriptional and translational levels. The inhibition of miR-665 resulted in a marked increase in SDC1 protein and mRNA expression in aging VSMCs. The correlation analysis showed that serum miR-665 mRNA was negatively correlated with SDC1 in individuals with aging and EVA. Finally, silencing SDC1 also reversed the effects of lncRNA GAS5 on VSMC senescence.

As with the majority of studies, the design of the current study is subject to limitations. Because our study focuses on miR-665, the investigation on lncRNA GAS5 is insufficient. Additionally, there were few animal experimental results. Therefore, subsequent studies on lncRNA GAS5 and animal experiments are required in the future.

The results of this experiment provide a possible explanation: miR-665 regulates VSMC senescence by targeting SDC1, and lncRNA GAS5 regulates SDC1 expression in aging VSMCs through sponging miR-665. We have predicted and confirmed that the lncRNA GAS5/miR-665/SDC1 axis is a crucial signaling pathway in VSMC senescence. Further investigations, including clinical trials, are needed to validate our results. Our findings may provide a new treatment strategy for aging-related CVD, such as atherosclerosis and hypertension.

## Data Availability Statement

The datasets presented in this study can be found in online repositories. The names of the repository/repositories and accession number(s) can be found at: GEO Series GSE174451.

## Ethics Statement

The studies involving human participants were reviewed and approved by Clinical Research Ethics Committee of the First Affiliated Hospital of Guangdong Pharmaceutical University. Written informed consent for participation was not required for this study in accordance with the national legislation and the institutional requirements. The animal study was reviewed and approved by the Institutional Animal Care and Use Committee of Guangdong Pharmaceutical University.

## Author Contributions

LZ and TC contributed to conception and design of the study. TC and QL performed the main experiments. JX, YaZ, YiZ, and LM performed the additional experiments. LZ, TC, and QL wrote the manuscript. All authors contributed to manuscript revision, read, and approved the submitted version.

## Conflict of Interest

The authors declare that the research was conducted in the absence of any commercial or financial relationships that could be construed as a potential conflict of interest.

## Publisher's Note

All claims expressed in this article are solely those of the authors and do not necessarily represent those of their affiliated organizations, or those of the publisher, the editors and the reviewers. Any product that may be evaluated in this article, or claim that may be made by its manufacturer, is not guaranteed or endorsed by the publisher.
