# Supplementary material for: MiR-665 Regulates Vascular Smooth Muscle Cell Senescence by Interacting With LncRNA GAS5/SDC1
Source: Front Cell Dev Biol. 2021 Jul 27;9:700006. doi: 10.3389/fcell.2021.700006 (PMC8353444; doi:10.3389/fcell.2021.700006)
Supplement: Supplementary file 1 [file Table_1.DOC]

| **MIRNA** | Forward Sequences (5 'to 3') | Reverse Sequences (5 'to 3') |
| --- | --- | --- |
| [hsa-miR-4742-3p](http://www.mirbase.org/cgi-bin/mirna_entry.pl?acc=hsa-miR-4742-3p) | ACACTCCAGCTGGGTCTGTATTCTCCTTTGCCT | CTCAACTGGTGTCGTGGAGTCGGCAATTCAGTTGAGCTGCAGG |
| [hsa-miR-433-5p](http://www.mirbase.org/cgi-bin/mirna_entry.pl?acc=hsa-miR-433-5p) | ACACTCCAGCTGGGTACGGTGAGCCTGTCATT | CTCAACTGGTGTCGTGGAGTCGGCAATTCAGTTGAGGAATAAT |
| [hsa-miR-376b-5p](http://www.mirbase.org/cgi-bin/mirna_entry.pl?acc=hsa-miR-376b-5p) | ACACTCCAGCTGGGCGTGGATATTCCTTCTAT | CTCAACTGGTGTCGTGGAGTCGGCAATTCAGTTGAGAAACATA |
| [hsa-miR-376b-3p](http://www.mirbase.org/cgi-bin/mirna_entry.pl?acc=hsa-miR-376b-3p) | ACACTCCAGCTGGGATCATAGAGGAAAATCCA | CTCAACTGGTGTCGTGGAGTCGGCAATTCAGTTGAGAACATGG |
| [hsa-miR-668-3p](http://www.mirbase.org/cgi-bin/mirna_entry.pl?acc=hsa-miR-668-3p) | ACACTCCAGCTGGGTGTCACTCGGCTCGGCCCA | CTCAACTGGTGTCGTGGAGTCGGCAATTCAGTTGAGGTAGTGG |
| [hsa-miR-6868-3p](http://www.mirbase.org/cgi-bin/mirna_entry.pl?acc=hsa-miR-6868-3p) | ACACTCCAGCTGGGTTCCTTCTGTTGTCTGT | CTCAACTGGTGTCGTGGAGTCGGCAATTCAGTTGAGCTGCACA |
| [hsa-miR-431-3p](http://www.mirbase.org/cgi-bin/mirna_entry.pl?acc=hsa-miR-431-3p) | ACACTCCAGCTGGGCAGGTCGTCTTGCAGGGC | CTCAACTGGTGTCGTGGAGTCGGCAATTCAGTTGAGAGAAGCC |
| [hsa-miR-1262](http://www.mirbase.org/cgi-bin/mirna_entry.pl?acc=hsa-miR-1262) | ACACTCCAGCTGGGATGGGTGAATTTGTAGAA | CTCAACTGGTGTCGTGGAGTCGGCAATTCAGTTGAGATCCTTC |
| [hsa-miR-487b-5p](http://www.mirbase.org/cgi-bin/mirna_entry.pl?acc=hsa-miR-487b-5p) | ACACTCCAGCTGGGGTGGTTATCCCTGTCCTG | CTCAACTGGTGTCGTGGAGTCGGCAATTCAGTTGAGCGAACAG |
| hsa-miR-3173-5p | ACACTCCAGCTGGGTGCCCTGCCTGTTTTCTC | CTCAACTGGTGTCGTGGAGTCGGCAATTCAGTTGAGAAAGGAG |
| hsa-miR-221-3p | ACACTCCAGCTGGGAGCTACATTGTCTGCTGGG | CTCAACTGGTGTCGTGGAGTCGGCAATTCAGTTGAGGAAACCC |
| hsa-miR-27b-3p | ACACTCCAGCTGGGTCACAGTGGCTAAGTT | CTCAACTGGTGTCGTGGAGTCGGCAATTCAGTTGAGGCAGAAC |
| hsa-miR-16-1-3p | ACACTCCAGCTGGGCCAGTATTAACTGTGCTG | CTCAACTGGTGTCGTGGAGTCGGCAATTCAGTTGAGTCAGCAG |
| hsa-miR-548k | ACACTCCAGCTGGGAAAAGTACTTGCGGATTT | CTCAACTGGTGTCGTGGAGTCGGCAATTCAGTTGAGAGCAAAA |
| hsa-miR-222-5p | ACACTCCAGCTGGGCTCAGTAGCCAGTGTAGA | CTCAACTGGTGTCGTGGAGTCGGCAATTCAGTTGAGAGGATCT |
| hsa-miR-1908-5p | ACACTCCAGCTGGGCGGCGGGGACGGCGATT | CTCAACTGGTGTCGTGGAGTCGGCAATTCAGTTGAGGACCAAT |
| hsa-miR-29b-1-5p | ACACTCCAGCTGGGGCTGGTTTCATATGGTGGTT | CTCAACTGGTGTCGTGGAGTCGGCAATTCAGTTGAGTCTAAAC |
| hsa-miR-3158-3p | ACACTCCAGCTGGGAAGGGCTTCCTCTCTGCA | CTCAACTGGTGTCGTGGAGTCGGCAATTCAGTTGAGGTCCTGC |
| hsa-miR-125b-1-3p | ACACTCCAGCTGGGACGGGTTAGGCTCTTGGG | CTCAACTGGTGTCGTGGAGTCGGCAATTCAGTTGAGAGCTCCC |

**Supplementary Table 1 Primer sequence**
